# Supplementary material for: Extreme isotopic heterogeneity in Samoan clinopyroxenes constrains sediment recycling
Source: Nat Commun. 2021 Feb 23;12:1234. doi: 10.1038/s41467-021-21416-9 (PMC7902626; doi:10.1038/s41467-021-21416-9)
Supplement: Supplementary file 1 — Supplementary Information [file 41467_2021_21416_MOESM1_ESM.pdf]

Supplementary Information

Extreme isotopic heterogeneity in Samoan clinopyroxenes constrains sediment recycling

Jenna V. Adams, Matthew G. Jackson, Frank J. Spera, Allison A. Price, Benjamin L. Byerly, Gareth Seward, and John M. Cottle

Supplementary Figures

Supplementary Figure 1: Location of Savai'i island, western Samoa and the location of the samples discussed in this study. ALIA-D115 lavas were dredged off the coast of Savai'i Island at 3,220 m water depth (at the ALIA-D115 dredge sample site denoted with a red dot; -172.896, -14.090)[1]. Map was made with the Generic Mapping Tools[2] using SRTM15+ elevation data[3,4].

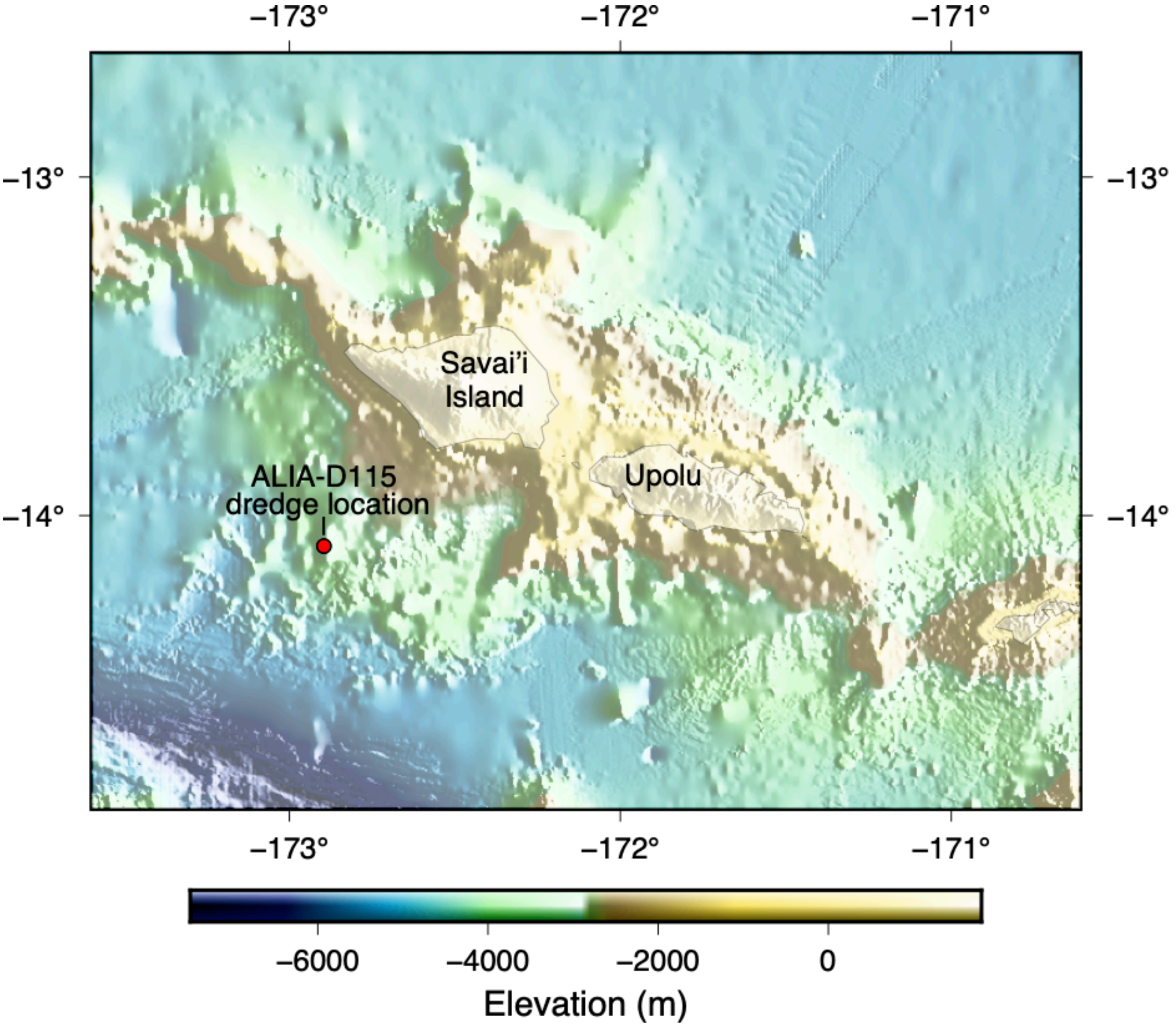

Supplementary Figure 2: Theoretical and model mixing calculations in relation to other Samoan whole rock lavas. The black hatched line is the theoretical mixing curve calculated using binary mixing theory (see Methods for details) and each hatch occurs in 10% mixing increments. The green star denotes the silicic, high $^{87}Sr/^{86}Sr$ inferred endmember composition from one of the binary mixing calculations. The grey line is a Magma Chamber Simulator (MCS) magma mixing simulation that incorporates the thermodynamics of combined magma mixing and fractional crystallization processes between the calculated mixing endmembers of this study. The mixing line represents the melt evolution as a result of mixing and crystallization (where crystals are removed from the system), thus mass is not conserved (unlike theoretical binary mixing) and the mixing line is not expected to end at the inferred EM2 endmember (see Methods for more detail). Since the grey line represents a real thermodynamically constrained simulation, in this case the simulation terminated when the melt reached ~63% crystallization meaning no solution could be found past this point. This highlights the differences between using simple binary mixing calculations that do not take into account thermodynamics whereas the MCS simulation, although not perfect, represents a more realistic view of these open system processes. The red squares represent the ALIA-D115 whole rock lavas, orange diamonds represent other Samoan whole rock lavas, and the purple circles are Samoan pillow glasses. See main text Figure 1 and 2 captions for data references.

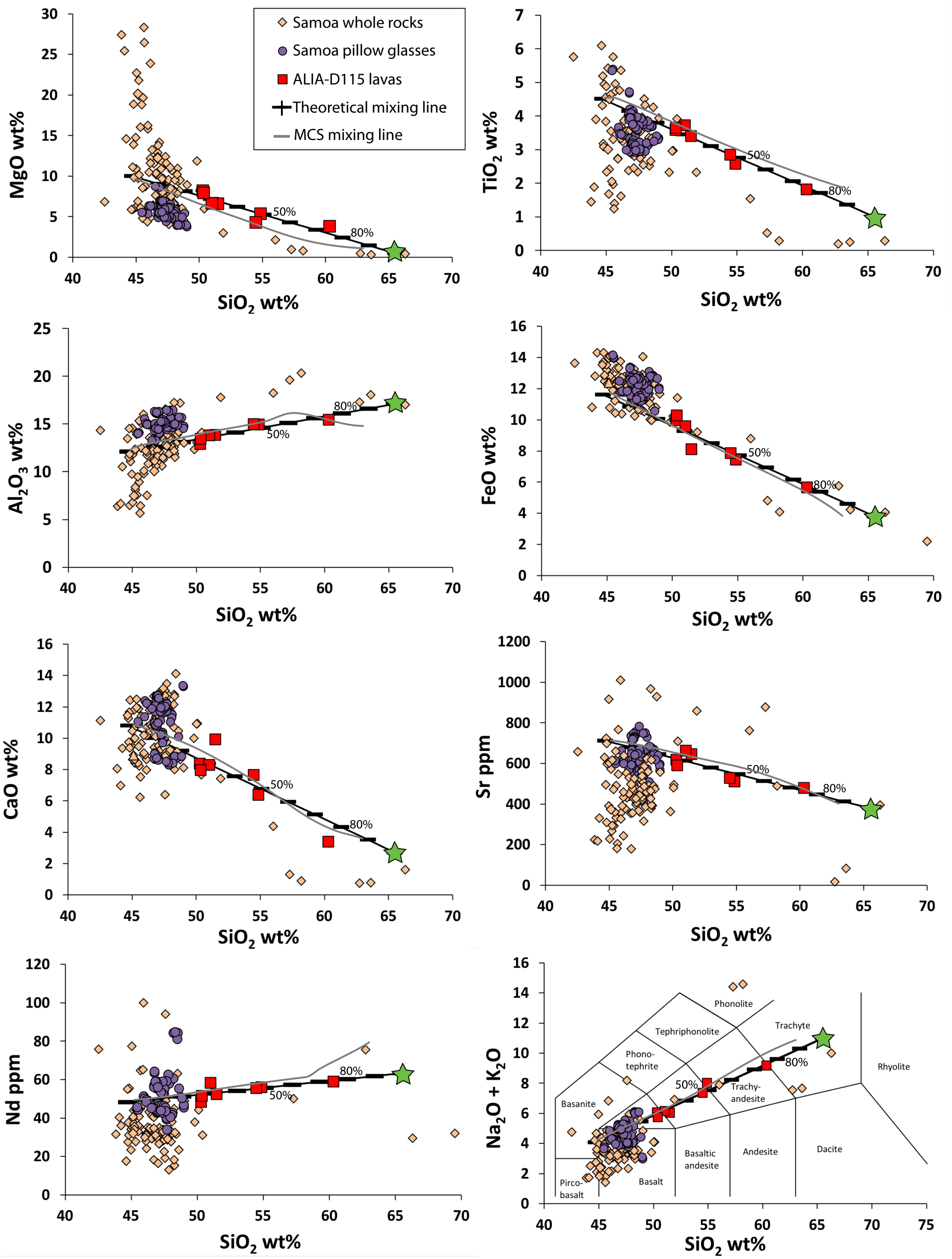
Samoa whole rocks
Samoa pillow glasses
ALIA-D115 lavas
Theoretical mixing line
MCS mixing line
MgO wt%
TiO_2 wt%
Al_2O_3 wt%
FeO wt%
CaO wt%
Sr ppm
Nd ppm
$Na_2O + K_2O$
SiO_2 wt%
50%
80%
Phonolite
Tephriphonolite
Trachyte
Phono-
tephrite
Basanite
Trachy-
andesite
Rhyolite
Basaltic
andesite
Andesite
Dacite
Pirco-
basalt
Basalt

Supplementary Figure 3: Theoretical and model mixing calculations in relation to other Samoan whole rock lavas in $^{87}Sr/^{86}Sr$ vs. element concentration space. Mixing is theoretically hyperbolic in ratio-element space. The green star denotes the composition of the silicic, high $^{87}Sr/^{86}Sr$ EM2-derived endmember and is compared with the ALIA-D115 whole rock (red squares) mixing trend, in addition to other Samoan whole rock lavas (orange diamonds) and Samoan pillow glasses (purple circles). See Supplementary Figure 2 caption for description of mixing lines and main text Figure 1 and 2 captions for data references.

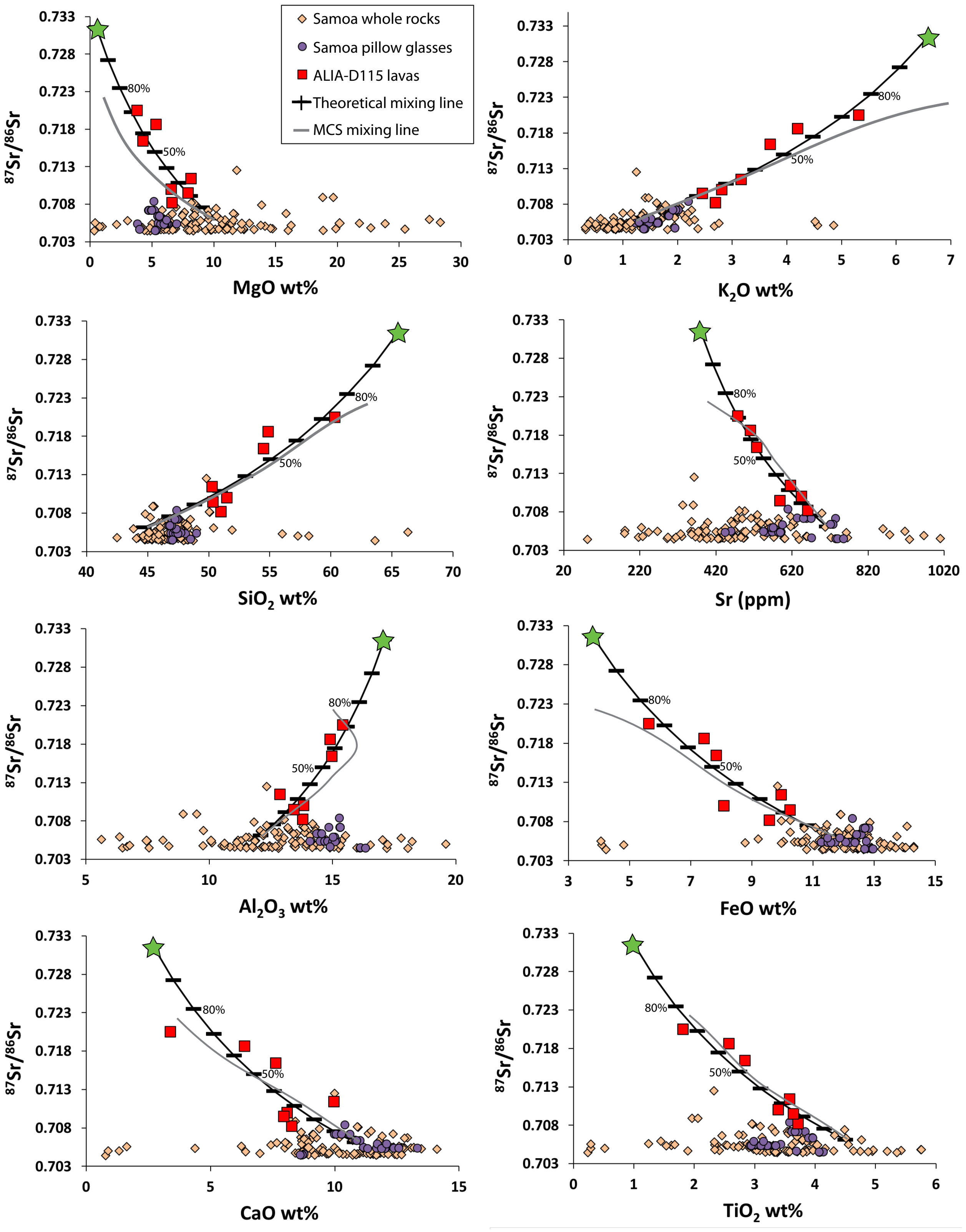

Samoa whole rocks
Samoa pillow glasses
ALIA-D115 lavas
Theoretical mixing line
MCS mixing line
87Sr/86Sr
0.733
0.728
0.723
0.718
0.713
0.708
0.703
80%
50%
MgO wt%
0 5 10 15 20 25 30
K2O wt%
0 1 2 3 4 5 6 7
SiO2 wt%
40 45 50 55 60 65 70
Sr (ppm)
20 220 420 620 820 1020
Al2O3 wt%
5 10 15 20
FeO wt%
3 5 7 9 11 13 15
CaO wt%
0 5 10 15
TiO2 wt%
0 1 2 3 4 5 6

Supplementary Figure 4: Ordinary least squares regression (ODR) for all ratio-ratio plots used to infer both the mafic (M), low $^{87}Sr/^{86}Sr$ and silicic (S), high $^{87}Sr/^{86}Sr$ mixing endmembers. The red squares are the ALIA-D115 whole rock lavas (*n*=7). The black line is the modeled regression fit which is of the form $\frac{C_2^H}{C_1^H} = A + \frac{B}{C_1^H}$, where C_1^H and C_2^H are the concentrations of chemical species 1 and 2 the mixed (**H**ybrid) magma. *A* can be thought of as the y-intercept and is of the form $A = \frac{C_2^M - C_2^S}{C_1^M - C_1^S}$ and *B* represents the slope given by the equation $B = \frac{(C_1^M C_2^S - C_1^S C_2^M)}{C_1^M - C_1^S}$, where C_1^M and C_2^M are concentrations of chemical species 1 and 2 in M and C_1^S and C_2^S are the concentrations in S. The *A* and *B* values are derived from the ODR, thus given *A* and *B* and the known value of MgO in C_1^M and C_1^S, then C_2^M and C_2^S can be calculated, given some re-arranging of the above equations (see Methods for more details), for all of the other major elements.

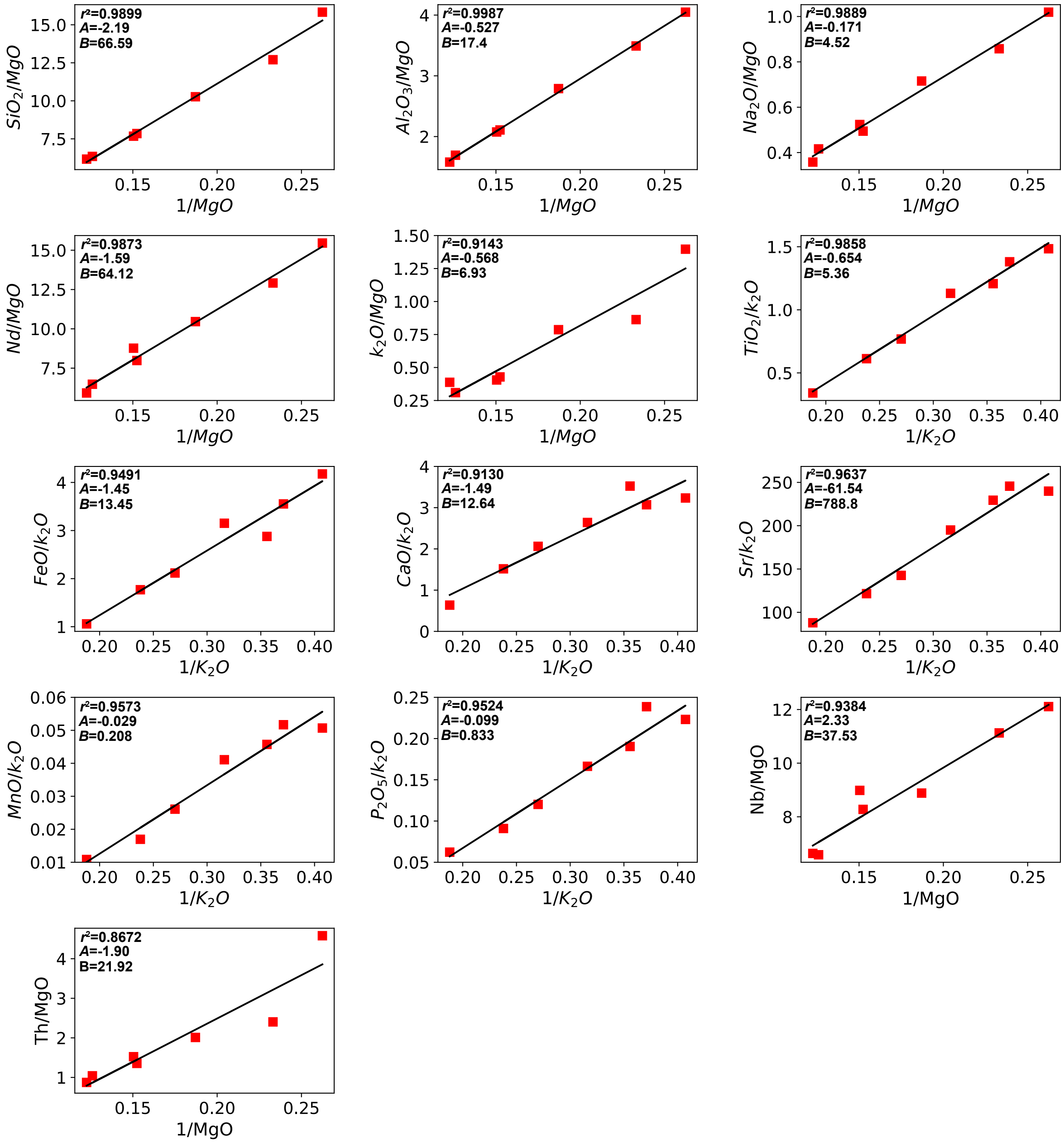

Supplementary Figure 5: Ordinary least squares regressions for isotope ratio vs. element ratio mixing plots. The red squares are the ALIA-D115 whole rocks (*n*=7). The black line is the modeled regression fit and the A and B values describe the equation for the fit – these values are summarized briefly in Supplementary Figure 4 and more thoroughly in the Methods section. The Sr and Nd concentrations used in these plots are from the regressions of Supplementary Figure 4. Thus, if the Sr and Nd concentrations are known, then using the equation for the linear regression described on isotope ratio versus element ratio space, one can calculate the $^{87}Sr/^{86}Sr$ and $^{143}Nd/^{144}Nd$.

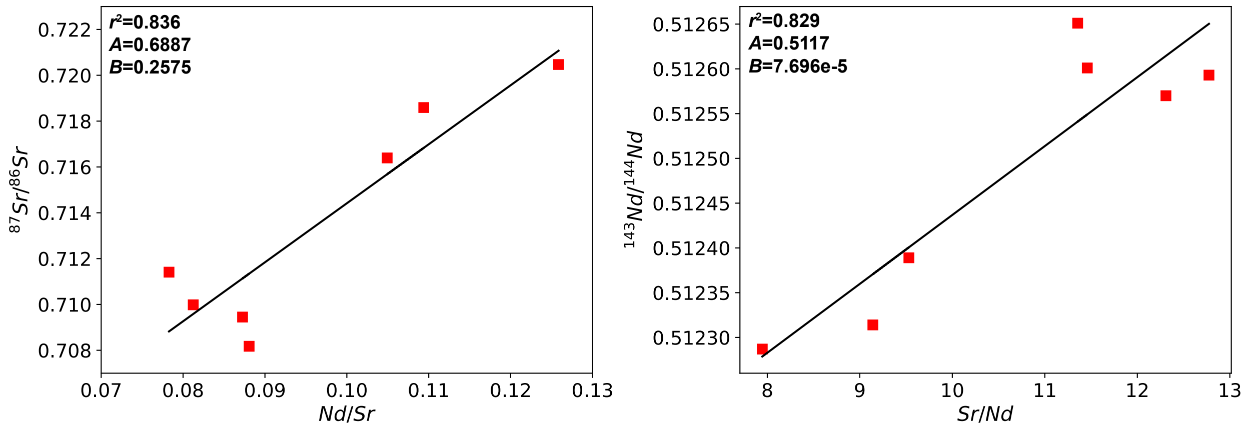

Supplementary Figure 6: Example of clinopyroxene major element oxide maps performed on the electron microprobe at UC Santa Barbara using 2 µm pixel size. Mg and Fe chemical zonation show irregular zoning patterns indicative of magmatic mixing and are also the fastest diffusing elements that have preserved chemical zoning in the ALIA-D115-18 clinopyroxenes (cpx). Mg-Fe inter-diffusion is particularly fast and the smallest compositional band thicknesses (lamella) are used to estimate magmatic residence times. Magma residence time calculations are described in detail in the Methods section. The smallest lamella thicknesses that exist in these clinopyroxenes are shown in the black box below measuring about 15 µm thick. The rainbow color scale for each image below indicates the weight percentage of either FeO (left) or MgO (right) with hot colors indicating higher percentages (up to 10 wt% for FeO and 22 wt% for MgO) and cold colors indicating lower percentages (down to 5 wt% for FeO and 8 wt% for MgO).

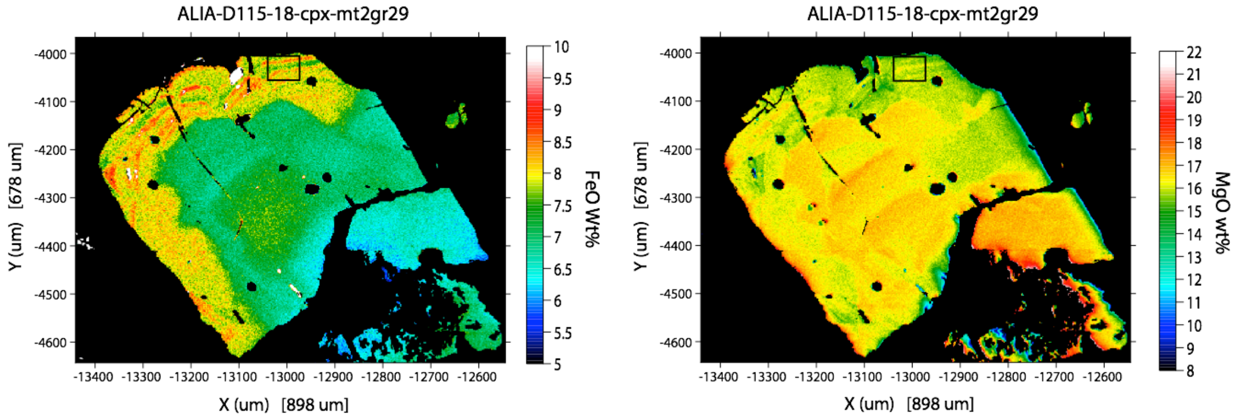

Supplementary Figure 7: Representative partial melting and fractional crystallization (FC) simulations of mixed peridotite+sediment bulk compositions. Simulations were performed using pMELTS[5] showing that the origin of the EM2-derived, trachytic mixing endmember calculated in this study likely does not originate from partial melting of a sediment contaminated peridotite source that subsequently undergoes differentiation. The differentiation of the partial melts consistently lead to phonolites, not trachytes (see Supplementary text for further discussion). The bulk starting mixture used for these simulations was MSCS-3G[6] sediment + peridotite with mixture proportions ranging from 5-20% MSCS-3G and 95-80% peridotite. The "X" symbols indicate 5% partial melt compositions, squares indicate 10% melt compositions, and circles indicate 20% partial melt compositions. The light grey line divides the 2 and 3 GPa partial melt simulations (3 GPa partial melts to the left of the line and 2 GPa partial melts to the right). Blue colors on either side of the grey line indicate a mixture of 5% MSCS-3G and 95% peridotite, reds indicate a 10% MSCS-3G and 90% peridotite mixture, and purples indicate a 20% MSCS-3G and 80% peridotite. In addition, 0.5 GPa FC simulations (dashed lines) using different 2 GPa partial melt compositions as starting compositions are plotted. Only the FC simulations performed on the partial melts performed at 2 GPa are shown as FC of the 3GPa partial melts look very similar. FC simulations were also run at 1 GPa and 0.3 GPa and show minute differences in their evolutionary paths, thus only the 0.5 GPa simulations are plotted for clarity. ALIA-D115 whole rock lavas (red squares) and other Samoan shield lavas (yellow diamonds) are also plotted for reference. Similar results as those presented below are found using any of the compositions outlined in Supplementary Table 3a under any of the parameterizations outlined in Supplementary Table 3b, thus only one set of simulations is shown below for clarity.

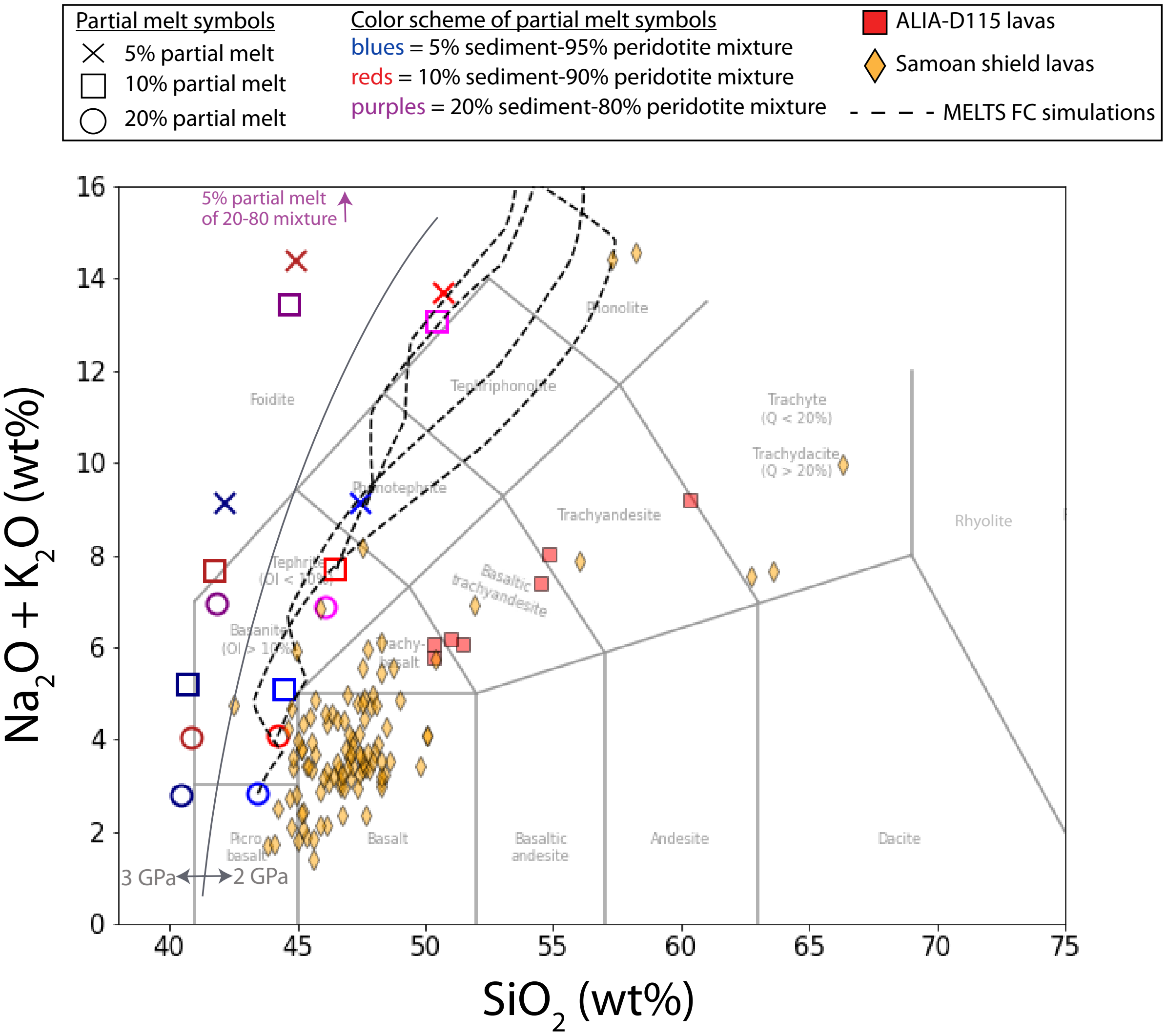

Supplementary Figure 8: Characteristic mantle source ratio, Nb/Th, versus $^{87}Sr/^{86}Sr$. Nb and Th values were calculated in this study in the two mixing endmembers, the mafic endmember (inferred mafic endmember; black star) and the trachytic EM2-derived endmember (inferred EM2-derived endmember; green star). These values are compared with other Samoan shield lavas (orange diamonds; all samples with less than 3 wt% MgO have been removed from the dataset) and the ALIA-D115 whole rock lavas (red squares). See main text Figure 1 for data references.

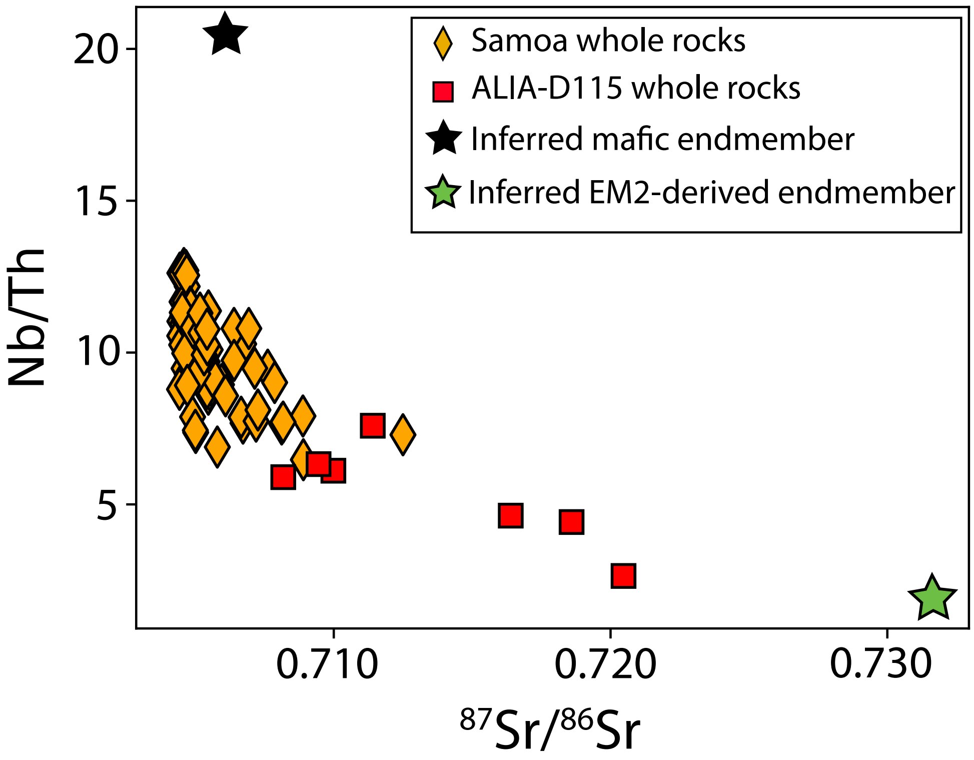

Supplementary Text

Supplementary note 1: Modeling the evolution of EM2 and the origin of trachytic liquids beneath Samoa

Following the magma mixing model proposed by recent work[7], the EM2 mixing endmember, proposed in this study to be trachytic in composition, is created by the generation of isotopically enriched, primitive mantle partial melts that are then differentiated to an evolved, and high $^{87}Sr/^{86}Sr$ magma that is then mixed with a more primitive, low $^{87}Sr/^{86}Sr$ magma to form the ALIA-D115 lava mixing trend. The final mixed magma may contain crystal cargo inherited

from either or both endmember magmas and, significantly, newly formed crystals can be the result of the mixing event itself. In this work, we take this model a step further by utilizing phase equilibria calculations to test this model. We suggest a scenario in which ancient subducted sediment (high in $^{87}Sr/^{86}Sr$) melts and fertilizes ambient peridotite to form a hybridized sediment + peridotite mixture. This mixture is subsequently partially melted at depths consistent with the lithosphere-asthenosphere boundary at Samoa, ~65 km (or 2-3 GPa) as well as Samoan mantle xenolith equilibration pressures of around 1-3.6 GPa using a Ca-in-olivine barometer[8] and slightly lower than the 3-5 GPa pressures obtained using the clinopyroxene-thermobarometry[9] These melts are then transported to shallow levels in the lithosphere (≤ 1GPa) where they undergo fractionation.

Supplementary note 2: Model parameterization and results

Roughly 40 simulations were carried out using pMELTS[5] and the Magma Chamber Simulator[10] (MCS) to simulate the partial melting and fractionation described above (see Supplementary Table 3a and b for starting compositions and model parameterizations, respectively). Partial melting from 5-20% equilibrium melting was carried out at 2 and 3 GPa on various bulk compositions (mixed sediment + peridotite bulk composition with the sediment being either MSCS-3G[6] or EPSM[11]). Regardless of starting composition, at 2 and 3 GPa, the partial melts range from phonolites at low degree partial melts (5%) to dominantly basanitic at high degree partial melts (15-20%) with one partial melt composition reaching a picro-basalt. The 2 GPa partial melts are consistently more silicic by ~3 wt% than the 3 GPa partial melts, but their alkali contents seem to remain constant between the 2 and 3GPa simulations. Fractional crystallization of these partial melts consistently leads to phonolitic melts (see Supplementary Figure 7 for an example partial melting and fractionation calculation). The existence of evolved,

low $^{87}Sr/^{86}Sr$ trachyte lavas erupted in Samoa show that highly differentiated magmas exist beneath Samoa and thus, EM2-like melts may undergo significant fractionation prior to mixing, but results from the above simulations are not consistent with the origin of the evolved, high $^{87}Sr/^{86}Sr$ trachytic liquids at Samoa being from partial melting of sediment contaminated peridotite followed by differentiation since we could only generate phonolitic to basanitic melts, not trachytic melts. Trachytic liquids are often thought to be derived through fractionation of alkali basalts[12–16]. In the case of Samoa, the radiogenic nature of the EM2 mixing endmember requires a sediment component in the source and this sediment addition inevitably adds alkalis to the system. Thus, partial melting sediment-contaminated peridotite results in melts more enriched in the alkalis compared with typical primitive mantle melts driving any fractionation of these liquids to phonolitic compositions, consistent with our phase equilibria calculations.

Supplementary note 3: Conclusions and future modeling

Bearing in mind the discussion above, the parameter space that can be explored in these calculations is quite large – the following parameters are important: pressure of melting, pressure of fractionation, starting bulk composition, fO_2, water content, type of melting (i.e., equilibrium versus incremental batch; where equilibrium melting involves partial melting in which the partial melt and solid are in complete equilibrium at all times and incremental batch melting involves achieving a certain melt fraction in stages, where specified fractions of melt are incrementally removed from the residual solid until the total desired melt fraction is melt), percentage of melting, to name the more salient ones. Considering these 7 variables, about 5000 models could be explored in this parameters space! Thus, further exploration of the model described above requires a Monte Carlo approach that we have not attempted, but should be explored.

References

1. Jackson, M. G. *et al.* The return of subducted continental crust in Samoan lavas. *Nature* **448**, 684–687 (2007).
2. Wessel, P., Smith, W. H. F., Scharroo, R., Luis, J. & Wobbe, F. Generic mapping tools: Improved version released. *Eos* **94**, 409–410 (2013).
3. Olson, C. J., Becker, J. J. & Sandwell, D. T. SRTM15_PLUS: Data fusion of Shuttle Radar Topography MIssion land topography with measured and estimated seafloor topography (NCEI Accession 0150537). *NOAA National Centers for Environmental Information* **Ver 1.1**, (2016).
4. Olsen, C. J., Becker, J. J. & Sandwell, D. T. A new global bathymetry map at 15 arcsecond resolution for resolving seafloor fabric: SRTM15_PLUS. in *American Geophysical Union 2014 Abstracts* (2014).
5. Ghiorso, M. S., Hirschmann, M., Reiners, P. & Kress, V. The pMELTS: A revision of MELTS for improved calculation of phase relations and major element partitioning related to partial melting of the mantle to 3 GPa. *Geochemistry Geophysics Geosystems* **3**, (2002).
6. Zhang, Y., Wang, C., Zhu, L., Jin, Z. & Li, W. Partial Melting of Mixed Sediment-Peridotite Mantle Source and Its Implications. *Journal of Geophysical Research: Solid Earth* **124**, 6490–6503 (2019).
7. Edwards, M. A. *et al.* Extreme enriched and heterogeneous ^{87}Sr/^{86}Sr ratios recorded in magmatic plagioclase from the Samoan hotspot. *Earth and Planetary Science Letters* **511**, (2019).
8. Hauri, E. H. & Hart, S. R. Constraints on melt migration from mantle plumes: a trace element study of peridotite xenoliths from Savai'i, Western Samoa. *Journal of Geophysical Research* **99**, (1994).
9. Putirka, K. D. Thermometers and barometers for volcanic systems. *Reviews in Mineralogy and Geochemistry* **69**, 61–120 (2008).
10. Bohrson, W. a. *et al.* Thermodynamic Model for Energy-Constrained Open-System Evolution of Crustal Magma Bodies Undergoing Simultaneous Recharge, Assimilation and Crystallization: the Magma Chamber Simulator. *Journal of Petrology* **0**, (2014).
11. Spandler, C., Hammerli, J. & Yaxley, G. M. An experimental study of trace element distribution during partial melting of mantle heterogeneities. *Chemical Geology* **462**, 74–87 (2017).
12. Le Roex, A. P. Geochemistry, mineralogy and magmatic evolution of the basaltic and trachytic lavas from Gough Island, South Atlantic. *Journal of Petrology* **26**, 149–186 (1985).
13. Cousens, B.L., Clague, D.A., and Sharp, W. D. Chronology, chemistry, and origin of trachytes from Hualalai Volcano, Hawaii. *Geochemistry Geophysics Geosystems* **4**, (2003).
14. Renzulli, A. & Santi, P. Two-stage fractionation history of the alkali basalt-trachyte series of Sete Cidades volcano (Sao Miguel Island, Azores). *European Journal of Mineralogy* **12**, 469–494 (2000).
15. Thompson, G., Smith, I. & Malpas, J. Origin of oceanic phonolites by crystal fractionation and the problem of the Daly gap: An example from Rarotonga. *Contributions to Mineralogy and Petrology* **142**, 336–346 (2001).
16. Berger, J., Ennih, N., Mercier, J.-C. C., LiéGeois, J.-P. & Demaiffe, D. The role of fractional crystallization and late-stage peralkaline melt segregation in the mineralogical

evolution of Cenozoic nephelinites/phonolites from Saghro (SE Morocco). *Mineralogical Magazine* **73**, 59–82 (2009).
17. Jochum, K.P., Weis, U., Stoll, B., Kuzmin, D., Tang, Q., Raczek, I., Jacob, D.E., Stracke, A., Birbaum, K., Frick, D.A., Gunther, D., Enzweiler, J. Determination of reference values for NIST SRM 610-617 glasses following ISO guidelines. *Geostandards and Geoanalytical Research* **35**, 397-429 (2011).
