# Supplementary material for: Extreme isotopic heterogeneity in Samoan clinopyroxenes constrains sediment recycling
Source: Nat Commun. 2021 Feb 23;12:1234. doi: 10.1038/s41467-021-21416-9 (PMC7902626; doi:10.1038/s41467-021-21416-9)
Supplement: Supplementary file 3 — Description of Additional Supplementary Files [file 41467_2021_21416_MOESM3_ESM.pdf]

Description of Additional Supplementary Files

File name: Supplementary Data 1
Description: Sr and Nd isotope TIMS data for individual clinopyroxenes from the ALIA-115-18 lava, in addition to major (EPMA) and trace element (LA-ICP-MS) data.

File name: Supplementary Data 2
Description: Calculated magma mixing endmember compositions (major and trace elements and Sr and Nd isotopes), at various starting MgO contents, and example clinopyroxene-hosted melt inclusion compositions.

File name: Supplementary Data 3
Description: Sediment + peridotite starting bulk compositions used for partial melting simulations utilizing the pMELTS thermodynamic modeling software

File name: Supplementary Data 4
Description: Trace element analysis and estimated precision of NIST612 glass by LA-ICP-MS performed during the clinopyroxene trace element analysis.
